# Discrepancy Between Surface Wear and Subsurface Fatigue Damage in CAD/CAM Composite Crowns: A Comparative Study of Intraoral Scans and Optical Coherence Tomography

**DOI:** 10.3390/dj14020084

**Published:** 2026-02-03

**Authors:** Julie-Jacqueline Kuhl, Maximiliane Amelie Schlenz, Bernd Wöstmann, Christin Grill, Ralf Brinkmann, Christoph Moos

**Affiliations:** 1 Department of Prosthodontics, University Hospital Schleswig-Holstein, Campus Kiel, Christian Albrecht University of Kiel, Arnold-Heller-Str. 3, 24105 Kiel, Germany; maximiliane.schlenz-helmke@uksh.de; 2 Department of Prosthodontics, Justus Liebig University Giessen, Schlangenzahl 14, 35392 Giessen, Germany; bernd.woestmann@dentist.med.uni-giessen.de; 3 Medical Laser Center Lübeck, Peter-Monnik-Weg 4, 23562 Lübeck, Germany; ch.grill@uni-luebeck.de (C.G.); ralf.brinkmann@uni-luebeck.de (R.B.); 4 Institute of Biomedical Optics, University of Lübeck, Peter-Monnik-Weg 4, 23562 Lübeck, Germany

**Keywords:** optical coherence tomography, CAD/CAM materials, fatigue materials, dental digital imaging, cracks, dental restoration

## Abstract

**Objectives:** This study aimed to determine whether surface wear, identified through the superimposition of intraoral scans (IOS), can predict subsurface damage progression detected by optical coherence tomography (OCT) during fatigue testing of computer-aided design/computer-aided manufacturing (CAD/CAM) composite crowns. **Methods:** Monolithic CAD/CAM composite crowns (Brilliant Crios; n=8) were adhesively luted to standardized prepared human teeth and artificially aged by cyclic loading in a mouth-motion simulator (50–500 N, 2 Hz, 37 °C). Under phantom-head condition, IOS (surface wear) and handheld swept-source (SS)-OCT (subsurface damage) were performed before loading and after every 250,000 cycles. OCT crack depth/width were normalized to local thickness and cusp-tip distance; correspondence between IOS- and OCT-derived metrics at each timepoint was assessed with Spearman’s rank correlation coefficient (ρ) to test whether surface wear can predict subsurface damage under the given conditions. **Results:** All specimens survived without catastrophic failure, and both modalities revealed progressive damage from the earliest observation interval. OCT consistently showed higher defect percentages and larger dispersion (e.g., mean vertical defects (25.47 ± 4.97)% OCT vs. (4.36 ± 0.91)% IOS at T1 and (66.79 ± 19.53)% OCT vs. (7.78 ± 3.19)% IOS at T5). Across all timepoints, no statistically significant associations between IOS and OCT were observed (*p* = 0.146 to 0.955). **Conclusions:** Within the limitations of this exploratory, single-material in vitro study, restricted to a CAD/CAM composite (Brilliant Crios), surface-based monitoring alone did not reliably reflect subsurface damage progression. Clinically, this suggests that surface wear assessment may underestimate subsurface fatigue damage. Intraoral OCT may provide complementary, non-invasive information alongside routine IOS for individualized monitoring, but its added value needs to be confirmed in larger studies and other CAD/CAM composite materials and additional restorative material classes.

## 1. Introduction

In current clinical workflows, monitoring of computer-aided design/computer-aided manufacturing (CAD/CAM) restorations relies mainly on superimposition of intraoral scans (IOS), which quantify surface wear but cannot detect subsurface fatigue damage [1,2]. This limitation motivates adjunct imaging capable of detecting subsurface defects under simulated, clinical-close conditions.

CAD/CAM technology enables the use of prefabricated industrial blocks with improved material homogeneity and standardized mechanical properties [3,4]. In addition, CAD/CAM composites offer several advantages in this context, such as ease of milling [5] and, in many systems, the possibility of intraoral repair.

From a mechanical perspective, failures in tooth-coloured restorative materials are largely associated with repetitive subcritical loading [6]. Fatigue damage typically originates under the occlusal contact area and progresses through crack initiation, propagation, and eventual partial or total failure [7,8,9]. Conventional analytical techniques, such as sectioning followed by light or scanning electron microscopy, are destructive and only permit post-test evaluation [10,11,12]. Although surface changes can be recorded by high-speed imaging [13,14], subsurface damage within tooth-colored materials remains inaccessible by these methods. Conventional clinical tools, including visual/tactile inspection, radiography and IOS-based surface analysis, likewise do not reliably reveal subsurface cracks in crowns, and radiography involves ionizing radiation and is not suitable for repeated fatigue monitoring.

OCT has emerged as a powerful non-invasive modality for depth-resolved imaging. Based on low-coherence interferometry, OCT combines backscattered sample light with a reference beam to enable cross-sectional visualization with micrometer-scale resolution [15,16,17].

OCT is widely used across medical disciplines and is gaining recognition in dentistry for its diagnostic potential [18]. Prior work has applied OCT to assess composite restorations [19,20,21], monitor polymerization processes [22], detect carious lesions [19,20], and visualize periodontal structures [23]. Data regarding OCT in prosthodontics is scarce and, in particular, OCT-based evidence for prosthetic restorative materials is comparatively limited [24,25,26,27]. Taken together, these applications highlight the potential of OCT to non-invasively detect subsurface defects that are not accessible by surface-based monitoring alone, making it particularly relevant for monitoring fatigue damage in CAD/CAM restorations [25]. Importantly, subsurface fatigue damage is clinically relevant, as crowns may exhibit wear facets without visible surface cracks while subsurface damage analysis still reveals internal cracks [28,29]. To the authors’ knowledge, only one study has investigated OCT-based monitoring of fatigue damage in different CAD/CAM materials [25]. However, this monitoring was performed in a laboratory set-up using a non-handheld OCT system that was not suitable for intraoral use, and no study has directly compared subsurface damage with surface wear under clinically realistic access, motivating the evaluation of chairside OCT for fatigue monitoring alongside routine IOS.

Despite this promise, the use of OCT for monitoring subsurface fatigue damage in monolithic CAD/CAM composite crowns remains insufficiently explored [24,25,26,27]. Establishing reliable OCT-based protocols for longitudinal monitoring would advance preventive dentistry by enabling timely interventions and by yielding insights into the long-term performance of composite materials and restoration designs. Such early detection of subsurface fatigue damage is clinically relevant because it may help prevent unexpected catastrophic crown fractures, avoid unplanned emergency treatment, and preserve tooth structure by enabling timely repair instead of full replacement. To demonstrate the feasibility of the proposed dual-modality monitoring workflow under controlled conditions, the present study was intentionally designed as a single-material investigation using a CAD/CAM composite.

This study establishes a reproducible chairside workflow that complements routine IOS with a handheld, intraoral dental OCT device to monitor subsurface damage propagation. Monolithic crowns were artificially aged in vitro by cyclic loading with simulated masticatory forces up to 500 N. Fatigue damage was monitored at predefined load cycle counts using IOS and dental OCT, and the correspondence between the two was quantified with matched vertical and horizontal metrics. The null hypothesis was that surface wear measured by IOS is not associated with subsurface damage propagation measured by OCT over time in this single-material setting.

## 2. Materials and Methods

### 2.1. Specimen Preparation

A total of n=8 specimens were prepared, following a three-step protocol: (i) caries-free human molars without restorations or visible cracks were collected with verbal informed consent under approval of the local ethics committee (Ref. No. 143/09), disinfected by immersion in 0.5% chloramine-T solution (Lysoform, Berlin, Germany) for six days in accordance with ISO/TS 11405 [30], then stored hydrated in distilled water; standardized abutments were prepared by computer-numerical controlled (CNC) milling under water cooling using a 5-axis high-speed milling machine (Mikron HSM 400, GF Machining Solutions GmbH, Schorndorf, Germany; diamond-coated grinding tools, spindle speed of approximately 50,000 rpm, feed rate 500 mm/min); the interval between tooth extraction and testing did not exceed six months; (ii) posterior crowns with fixed wall thickness and cement space were CAD/CAM-milled (CORiTEC 250i, imes-icore, Eiterfeld, Germany) from the resin-based composite Brilliant Crios (BC, Coltene, Altstätten, Switzerland) and polished; and (iii) the crowns and teeth were cleaned and conditioned, adhesively luted using a universal adhesive and dual-cure resin cement under a standardized seating force, followed by polymerization with a calibrated polywave LED curing unit. All specimens were stored in distilled water at 37 °C for ≥24 h before mouth-motion simulation, and were kept hydrated thereafter. This protocol was adapted from a previous study and is restricted here to BC; step-by-step specimen preparation is detailed in [7,25,31]. Exact materials and processing parameters are summarized in Table 1.

### 2.2. Artificial Aging: Mouth-Motion Simulator

**Figure 1:**
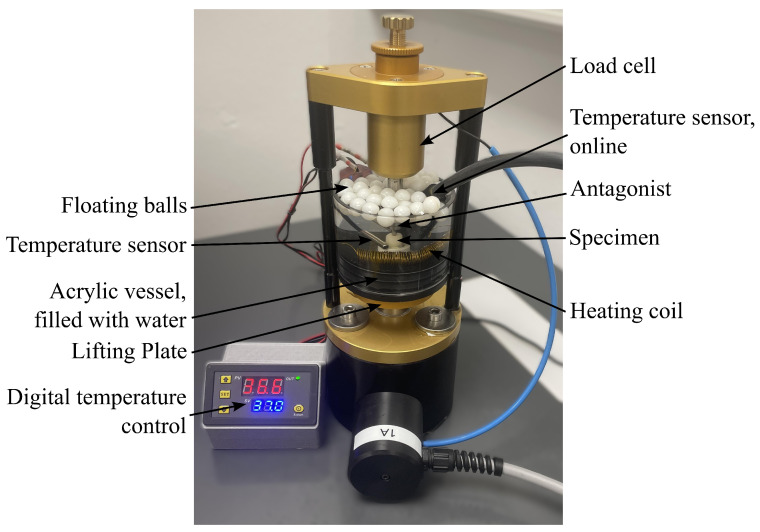
Artificial aging: mouth-motion simulator setup for cyclic loading.

Specimens were aged in a computer-controlled mouth-motion simulator (prematecF1000, wl-tec, Wertheim, Germany) under vertical cyclic loading between 50 N and 500 N at 2 Hz, see Figure 1. This load range represents physiologic posterior masticatory forces and includes an upper-range level relevant to parafunctional loading. The central fossa was loaded under single-point contact using a rounded stainless-steel antagonist (tip radius r = 1mm), representing a worst-case loading scenario. Mouth-motion simulation was performed in distilled water at a constant temperature of (37 ± 2) °C without thermocycling, as the study focused on a standardized IOS vs. OCT method comparison rather than full oral aging simulation. Temperature control used a resistive heating coil (ISOTAN, 2.5 Ω/m; Isabellenhütte Heusler, Dillenburg, Germany) powered by two dual-stabilized laboratory supplies (Model 6145, PeakTech, Ahrensburg, Germany) and regulated by a digital temperature controller (W3230, Aideepen, Shenzhen, China). Water level and temperature were monitored in real time with a sensor (ALL3418v2, Allnet, Germering, Germany). Floating balls were used to prevent rapid evaporation of the water. A total of 1,250,000 load cycles were applied, representing a clinical observation period of five years [12,32].

### 2.3. Monitoring

Dual-modality monitoring (IOS and OCT) was performed at baseline (T0), and after 250,000 (T1), 500,000 (T2), 750,000 (T3), 1,000,000 (T4), and 1,250,000 (T5) cycles in a mouth-motion simulator, with 250,000 cycles corresponding to approximately one year of intraoral function under average chewing conditions, based on the literature [32]. Measurement was performed under clinical-close conditions in a phantom head: IOS quantified surface wear, and handheld dental OCT assessed subsurface damage propagation. All acquisitions and measurements were performed by a single calibrated examiner; thus, inter-examiner reliability was not applicable. Matched vertical and horizontal metrics enabled direct comparison. Between chewing-simulation runs and imaging, specimens were kept continuously moist in distilled water.

**Figure 2:**
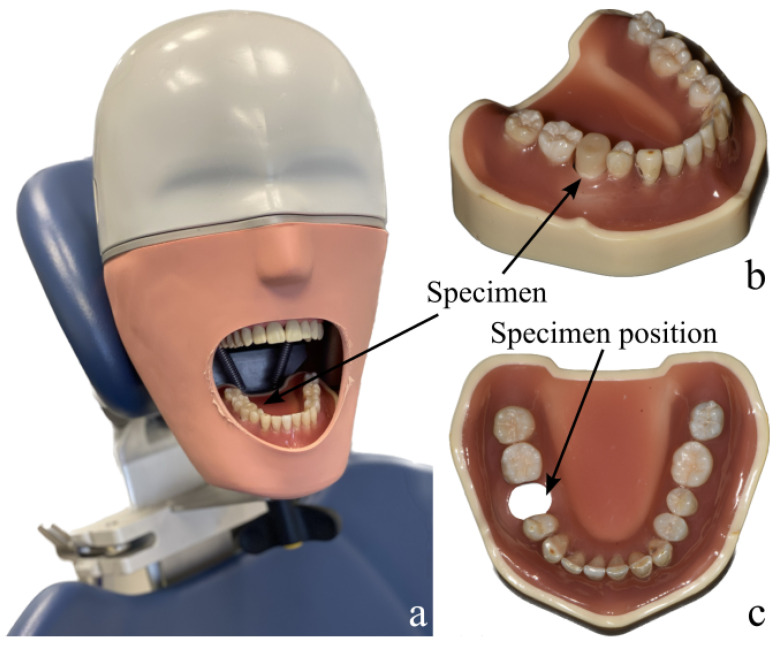
Phantom-head setup for dual-modality monitoring: Model mounted in a dental phantom head for simulating clinical-close conditions (**a**), human teeth model with specimen placed at tooth 45 position (**b**) and without specimen (**c**).

To approximate clinical conditions, a model composed of human teeth was fabricated prior to the study and mounted in a dental phantom head for IOS and OCT scanning (see Figure 2a). At the position of tooth 45 (FDI scheme), a basal placeholder allowed clamping of the specimens from below (see Figure 2b,c). Reproducible mounting was ensured by congruent basal alignment marks on the specimen holder and the model together with a dedicated insertion key, enabling identical positioning across specimens.

#### 2.3.1. Intraoral Scan: Surface Wear Monitoring

Quadrant scans were conducted with an intraoral scanner (Primescan AC, Dentsply Sirona, Bensheim, Germany; version CEREC SW 5.2.8) following a standardized pattern. To ensure comparable testing conditions, the same scanning path was applied for all scans. Scanning started on the occlusal surfaces, continued along the oral surfaces, and returned along the buccal surfaces until full surface coverage was achieved [33]. IOS data were analyzed in GOM Inspect Pro 2019 (Carl Zeiss GOM Metrology, Braunschweig, Germany), a 3D measurement software. For each specimen, scans from T1–T5 were rigidly registered to the T0 reference by best-fit alignment on unaltered neighboring anatomy adjacent to the occlusal contact area. Here, the occlusal contact area was defined as the area loaded by the antagonist in the central fossa. External surface wear was quantified as the maximum vertical and horizontal distance within the occlusal contact area between the T0 reference and each follow-up (T1–T5), normalized to the local occlusal thickness and cusp tip distance, respectively. For each load cycle (T1–T5), the arithmetic mean across specimens was reported.

#### 2.3.2. Optical Coherence Tomography: Subsurface Fatigue Damage Monitoring

**Figure 3:**
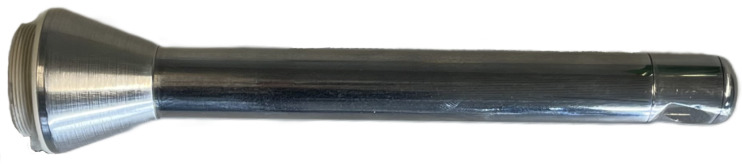
Handheld intraoral optical coherence tomography (OCT) device for volume imaging under clinically realistic access.

Non-invasive imaging was performed with a SS-OCT system (Vega VEG210C1, Thorlabs, Newton, NJ, USA) operated via ThorImage OCT (version 5). A custom handheld intraoral OCT device (optomechanical relay probe), developed for this study (Medical Laser Center Lübeck, Lübeck, Germany), was coupled to the SS-OCT system to adapt beam delivery and imaging geometry for intraoral access and enable volume imaging under clinically realistic access conditions (see Figure 3). A stainless-steel housing with sapphire windows (17221, Edmund Optics, York, UK) encloses a 30 mm focusing lens (45794, Edmund Optics, York, UK), two 25 mm relay lenses (45793 Edmund Optics, York, UK) and a folding mirror (MRA10-M01, Thorlabs, Newton, NJ, USA) that deflects the light beam. An integrated camera provided visual guidance, aided by an LED (powered by two external AA batteries, 3 V/20 mA). The intraoral OCT device was electrically isolated from the OCT scan head.

Mounted to the OCT scan head and configured for the SS-OCT’s 1300 nm center wavelength, the intraoral OCT device provides a 6 mm working distance, a maximum field of view of 8 mm × 8mm, and a maximum spatial resolution of 18 μm (axial) and 20 μm (lateral). OCT volume images covering the occlusal contact area were acquired with a 25 μL distilled-water droplet applied to the occlusal surface to reduce specular reflection and improve image contrast. The scan field was set to the maximum, 8 mm × 8 mm in X and Y and 11.03 mm in Z, covering the specimen to 11 mm depth from the occlusal surface. Given the optimal working distance of 6 mm, the occlusal contact area was positioned at approximately 5 mm in Z in the 2D preview to maintain focus across the full occlusal layer thickness.

**Figure 4:**
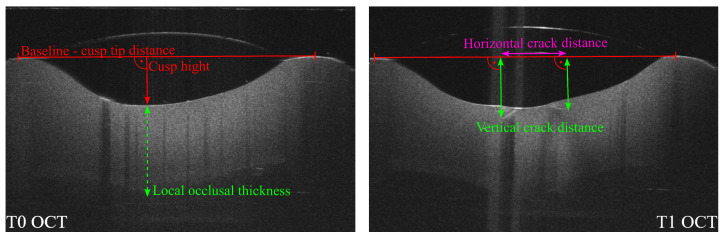
Representative optical coherence tomography (OCT) cross-sectional images illustrating the geometry-normalization used for quantitative crack assessment. **Left** (T0): determination of the cusp-tip distance (baseline), cusp height, and local occlusal thickness (reference geometry). **Right** (T1): measurement of the maximum vertical crack distance and maximum horizontal crack distance; vertical values were referenced to the baseline and normalized to the local occlusal thickness, and horizontal values were normalized to the cusp-tip distance (schematic overlays added for clarity). Timepoints: Baseline (T0) and after 250,000 (T1) load cycles.

Subsurface damage was quantified at T1–T5 as the deepest vertical crack penetration and the widest horizontal crack span relative to the local occlusal thickness and the cusp tip distance, respectively, using ThorImage measurement tools. For each timepoint, the OCT volume was reviewed at predefined depth levels, and the maximum vertical and horizontal crack extents were recorded. Measurements were performed using consistent anatomical reference landmarks (cusp tips and central fossa) across timepoints to standardize the vertical and horizontal measurements. A representative OCT cross-sectional image illustrating the geometry-normalization procedure is shown in Figure 4. For each load cycle, the arithmetic mean across specimens was reported. Although refractive-index calibration would in principle allow conversion of OCT optical path lengths to metric units, the required water droplet introduces case-specific lensing due to variable curvature, making absolute distances unreliable for this application. Therefore, subsurface damage is reported as relative percentages. In clinical use, absolute millimeter values are likewise less robust; instead, the clinically relevant information is the extent of damage (depth and width) relative to prominent anatomical landmarks within the crown.

### 2.4. Statistical Analysis

Descriptive statistics were computed for IOS- and OCT-derived metrics at five predefined assessment timepoints (T1–T5) and are reported as mean ± standard deviation and median (interquartile range). Given the exploratory single-material design, no a priori sample size or power calculation was performed. The sample size was set to n=8 in line with previous fatigue studies on CAD/CAM crowns using comparable mouth-motion simulator protocols, where similar specimen numbers are commonly employed [12,25,31,34]. Spearman’s rank correlation coefficient (ρ) was used because of the small sample size (n=8) and the exploratory nature of the analysis, which did not justify parametric correlation testing. Correlations were calculated between IOS- and OCT-derived vertical and horizontal metrics at each timepoint. Two-sided tests with α=0.05 were used; *p*-values are reported without adjustment for multiple comparisons (exploratory analysis). All analyses were performed in SPSS Statistics (version 29; IBM, Armonk, NY, USA).

## 3. Results

None of the specimens exhibited debonding or partial/complete failure after artificial aging. Nevertheless, fatigue damage was already detectable at T1 in all specimens.

**Figure 5:**
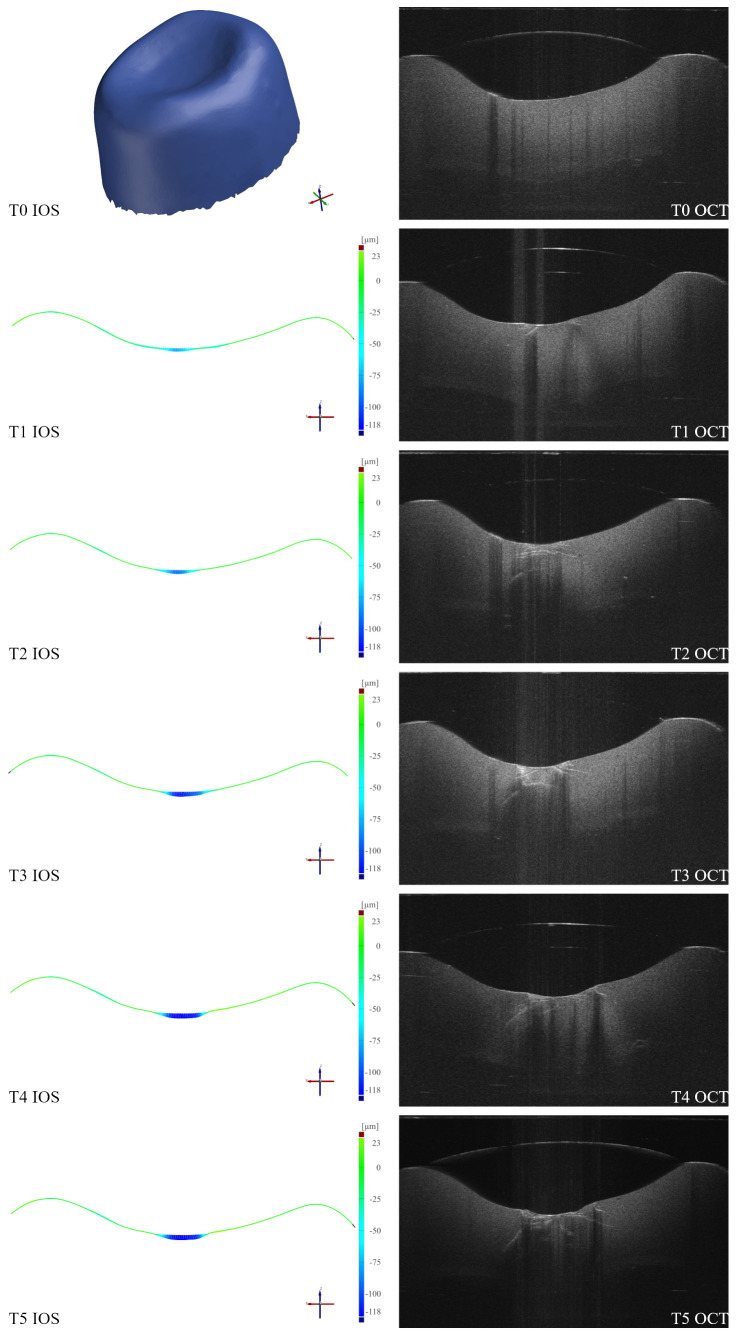
Comparison of surface wear and subsurface damage over time. (**Left**): Intraoral scans (IOS) cross-sectional deviation maps (T1–T5) referenced to T0, showing progressive external surface wear. (**Right**): Corresponding optical coherence tomography (OCT) sections (T0–T5) depicting deepening and widening of subsurface cracks. Timepoints: Baseline (T0), and after 250,000 (T1), 500,000 (T2), 750,000 (T3), 1,000,000 (T4), and 1,250,000 (T5) load cycles.

Figure 5 shows a cross-section through the occlusal contact area for one representative specimen across the load cycles T1–T5. In the IOS panel (left), each timepoint displays the T0-referenced deviation in the same cross-section, illustrating a progressive increase in vertical and horizontal surface wear from T1 to T5. In the OCT panel (right), consecutive OCT cross-sections of the occlusal contact area indicate a visual continuation (deepening and broadening) of subsurface defects from T1 to T5. While both modalities indicate progression, their patterns differ: IOS captures external surface wear, whereas OCT depicts subsurface damage propagation. Overall, both IOS and OCT revealed progressive damage over time, with OCT consistently indicating higher internal damage than would be expected from the surface wear alone.

**Figure 6:**
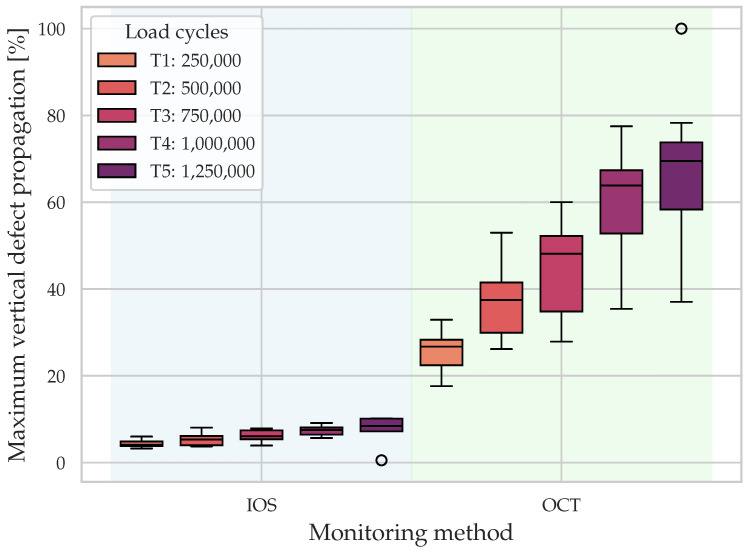
Maximum vertical defect propagation (%) by intraoral scans (IOS, surface wear) and optical coherence tomography (OCT, subsurface damage) across timepoints T1–T5. Boxplots illustrate progressive damage over time, with OCT yielding systematically higher values than IOS.

Figure 6 displays the maximum vertical defect propagation (%) of all specimen for IOS and OCT across T1–T5. Both modalities show a monotonic increase in central tendency with time, indicating progressive damage. OCT yields systematically higher values than IOS across all timepoints. This systematic difference can be explained by the fact that IOS measures external surface wear, whereas OCT identifies subsurface crack propagation that may occur without proportional surface material loss. At T5, one specimen exhibited full-thickness vertical cracking by OCT (approaching 100%) while showing minimal external surface wear by IOS, illustrating the potential dissociation between subsurface crack depth and surface wear (see Figure 6).

**Figure 7:**
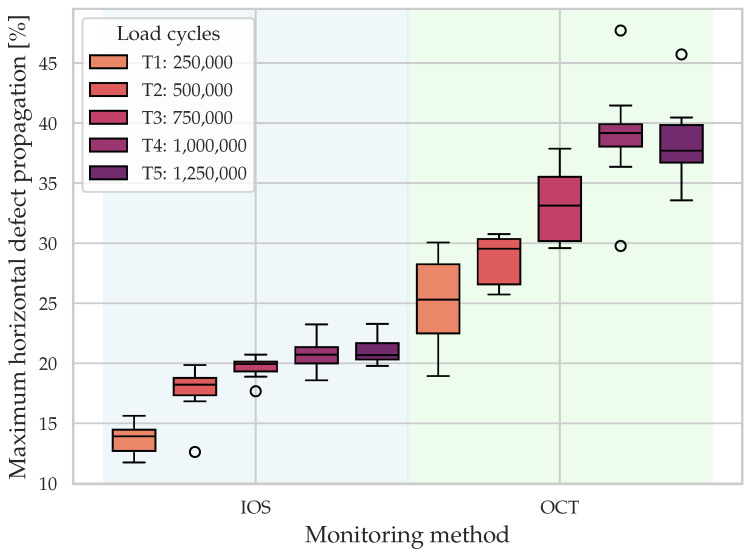
Maximum horizontal defect propagation (%) by intraoral scans (IOS, surface wear) and optical coherence tomography (OCT, subsurface damage) across timepoints T1–T5. Both modalities show progressive damage, with OCT yielding higher values than IOS, although between-method differences are smaller than for the vertical metrics.

Figure 7 displays the maximum horizontal defect propagation (%) of all specimen for IOS and OCT across T1–T5. Consistent with the vertical metrics, both modalities show a monotonic increase in central tendency over time, indicating progressive damage. OCT again yields systematically higher values than IOS across all timepoints. However, the between-method differences are smaller here than for the vertical metrics.

Numerical descriptive statistics (mean ± standard deviation and median (interquartile range)) for IOS- and OCT-derived vertical and horizontal metrics at all timepoints are provided in Table 2 and describe central tendency and dispersion without assuming normality. These data confirm the progressive increase in defect percentages over time for both modalities and show a comparable level of between-specimen variability across timepoints.

For each timepoint (T1–T5), Spearman’s correlation coefficient (ρ) was computed between vertical and horizontal surface wear (IOS) as well as vertical and horizontal subsurface damage propagation (OCT). As summarized in Table 3, none of the correlations reached statistical significance (all p>0.05), indicating no evidence of an association between IOS and OCT at any timepoint. As one T5 vertical OCT value appeared to be an extreme observation (approaching 100%), a sensitivity analysis was performed excluding this specimen from the T5 vertical analysis; the vertical IOS vs. OCT association at T5 remained non-significant (ρ=0.655, p=0.111, n=7). Accordingly, we failed to reject the null hypothesis that there is no association.

**Figure 8:**
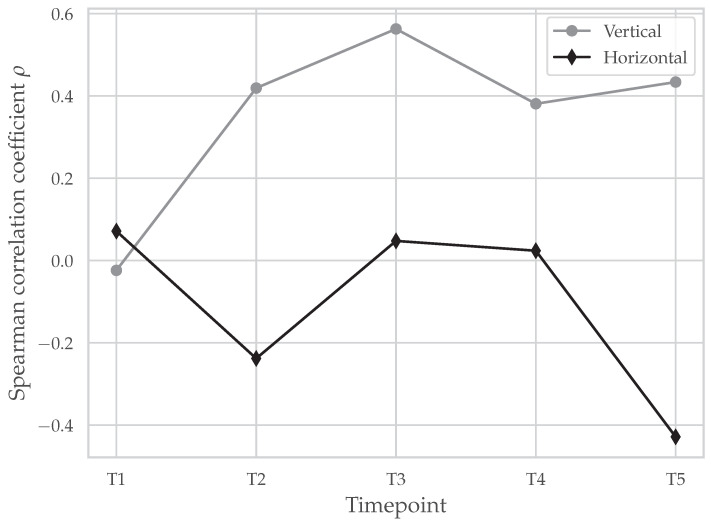
Progression of Spearman’s correlation coefficient (ρ) between surface wear and subsurface damage propagation across timepoints for vertical and horizontal metrics. Timepoints: After 250,000 (T1), 500,000 (T2), 750,000 (T3), 1,000,000 (T4), and 1,250,000 (T5) load cycles.

In Figure 8, Spearman’s correlation coefficients between the maximum vertical and horizontal metrics from IOS and OCT are plotted for T1–T5. Across assessment timepoints, effect sizes (following Cohen [35]) varied from small negative to medium and large positive values, yet all tests were non-significant (all *p* > 0.05). Together with Table 3, these findings provide no evidence of a reliable association between IOS and OCT at any timepoint. Accordingly, externally visible surface wear did not reliably predict subsurface damage progression under the present conditions, even though both modalities showed progressive damage over time.

## 4. Discussion

This exploratory single-material in vitro study showed that, under clinical-close dual-modality monitoring, externally visible surface wear assessed by IOS did not reliably reflect subsurface fatigue damage detected by OCT in CAD/CAM composite crowns. Using a reproducible, clinically inspired workflow that combines routine intraoral scanning with a handheld intraoral SS-OCT device in a dental phantom head, longitudinally surface wear and subsurface damage under cyclic loading was monitored. To the best of the authors’ knowledge, this is the first in vitro investigation to assess the correspondence between externally visible wear and subsurface fatigue damage in a chairside setup.

All specimens survived the aging protocol without catastrophic failure, yet both modalities revealed progressive damage from the earliest timepoint (T1). IOS captured monotonic increases in vertical and horizontal surface wear, while OCT visualized a deepening and widening of subsurface defects across T1–T5. Crucially, no statistically significant association between IOS and OCT at any timepoint was found (all p>0.05; Table 3). Thus, no evidence that surface wear reliably predicts subsurface damage progression under the present conditions was observed. The dissociation is exemplified by one specimen that showed full-thickness vertical cracking by OCT at T5 while exhibiting minimal external wear by IOS (see Figure 6).

Previous OCT studies have visualized internal defects and interfaces in vitro, but translation to clinically realistic, chairside monitoring has been hampered by access and scanning geometry. For example, Wendler et al. [36] quantified the remaining intact layer thickness of crowns rather than individual cracks, focusing on replacement thresholds. In contrast, the present approach localizes and tracks the extent of internal defects over time under clinical-close conditions. While internal degradation under cyclic loading is well documented, the present data emphasize that subsurface crack growth and external wear can follow different temporal profiles, underscoring the complementary nature of IOS and OCT [25].

Specimen preparation used standardized, CNC-milled abutments from human third molars to combine geometric reproducibility with realistic bonding to dentin, consistent with prior work on the advantages of human substrates for fatigue and fracture testing [12,37,38]. This workflow has been applied in a previous study [7,25,31]. While silicone keys and parallelometers can assist in standardizing preparations [11,12,39,40], only CAD/CAM milling ensured identical abutment geometry and crown configuration, which is critical given the influence of abutment form on restoration strength and stress distribution [41]. Adhesive luting followed manufacturer recommendations given its known benefits for fracture resistance over conventional cementation [7,11,31].

Various methods exist to simulate the masticatory loads experienced by dental restorations in vivo, though no system fully replicates all intraoral conditions [42]. Dynamic, cyclic loading systems are considered the most physiologically relevant, as they better approximate functional stress patterns [8,43]. Since clinical predictive power increases with loading duration, extended intervals over months or years are recommended [44]. Continuous antagonist–specimen contact was used to create a more severe cyclic loading regime than intermittent contacts during normal chewing, approximating a worst-case fatigue scenario relevant to parafunctional loading rather than a literal simulation of clinical bruxism.

To approximate functional loading, specimens were cyclically loaded between 50 N and 500 N in water at 37 ± 2 °C, covering normal masticatory forces in the lower range and extending into the upper range reported for patients with increased masticatory activity such as parafunctions [8,12,44]. This load range was chosen to represent an upper-end fatigue scenario for CAD/CAM composite crowns, which are often recommended for patients with higher masticatory forces. A single-point stainless-steel antagonist with continuous contact in the central fossa was used as a standardized laboratory fatigue indenter; its Vickers hardness (385 HV) approximates enamel (300 HV to 400 HV) while enabling a reproducible laboratory fatigue protocol rather than a literal replication of clinical occlusion [11,25,45]. All testing and storage were conducted in distilled water to reflect the moist intraoral environment, acknowledging moisture-assisted crack growth in tooth-colored materials [12,46,47].

During OCT, a distilled-water droplet reduced surface reflection on plane occlusal geometries, improving image contrast but introducing case-specific lensing; absolute scaling remains unreliable even with refractive-index correction [48]. Therefore, internal damage has been reported as relative percentages normalized to local occlusal thickness (vertical) and cusp-tip distance (horizontal), yielding clinically interpretable progression metrics without specimen-specific geometric calibration. This approach supports practical application, as defect areas can be reassessed during repair, with OCT offering a non-invasive, high-speed, and patient-friendly imaging method [49,50].

The association between IOS and OCT was assessed at each timepoint using Spearman’s correlation coefficient (ρ). All *p*-values were above 0.05 and effect sizes showed no consistent pattern, supporting the interpretation of no reliable association across the observed range. Statistical significance alone does not imply clinical usefulness; clinically meaningful predictability would require a consistently strong association across timepoints rather than isolated correlations. For current clinical practice, this implies that standard surface monitoring protocols should be interpreted with caution, as apparently minor occlusal changes do not exclude advanced internal fatigue damage. Under the present conditions, surface wear did not reliably reflect subsurface damage progression. Surface-based assessments alone may therefore underestimate clinically consequential subsurface damage. Intraoral OCT provides complementary, non-invasive information on subsurface damage that could, if confirmed in clinical studies, be selectively integrated into recall visits. In particular, repeated OCT imaging at recall could help to individualize recall intervals (e.g., shorter recalls for progressing OCT findings and routine recalls for stable findings) and support minimally invasive repair decisions—for example, by detecting progressing subsurface cracks beneath apparently stable surfaces and thereby prompting early repair instead of late fracture replacement. However, its added value for routine fatigue monitoring needs to be confirmed in larger in vitro and clinical studies.

Despite its clinically inspired setup, the following limitations must be acknowledged. First, the workflow was developed and evaluated using only a CAD/CAM composite. BC was selected as a clinically used CAD/CAM composite material, recently introduced yet well established, to serve as a suitable proof-of-concept material for this workflow, as it has demonstrated very good performance in previous studies [7,25,31,51]. As such, its applicability to other material classes including hybrid ceramics, silicate-based glass-ceramics, and oxide ceramics remains uncertain. Subsequent research should evaluate the transferability of the workflow across a broader range of CAD/CAM materials and restoration designs. In particular, hybrid ceramics, silicate-based glass-ceramics, and oxide ceramics exhibit different fracture toughness, crack propagation patterns, and OCT signal characteristics due to their higher stiffness, opacity, and refractive index, such that both fatigue behavior and detectability of subsurface defects may differ substantially from the present findings. Second, the in vitro phantom-head model cannot fully replicate the biological and mechanical complexity of the oral environment. The present setup does not reproduce patient-specific factors such as saliva and biofilm, head movement or thermal fluctuations, all of which may influence the clinical detectability of damage. Consequently, clinical studies are necessary to validate clinical feasibility, assess predictive accuracy, and evaluate long-term in vivo performance. Third, the sample size was limited (n=8) and no a priori power calculation was performed; the reported effect sizes and correlation estimates are therefore exploratory and should be interpreted with caution. Fourth, OCT imaging required a distilled-water droplet to optimize image contrast on plane occlusal geometries, which can introduce case-specific lensing. This motivated the use of geometry-normalized relative OCT metrics rather than absolute distances and represents a technical limitation of the current implementation.

## 5. Conclusions

Within the limitations of this exploratory, single-material in vitro study, dual-modality monitoring under clinical-close conditions revealed progressive surface wear and subsurface fatigue damage in CAD/CAM composite restorations. This dual-modality workflow advances the understanding of fatigue damage in CAD/CAM composite restorations by combining IOS-based surface wear analysis with OCT-based assessment of subsurface crack propagation under clinically inspired conditions. However, externally visible surface wear assessed by IOS did not reliably predict subsurface damage progression detected by OCT at any assessment timepoint. In this preliminary, single-material in vitro study, surface-based assessment alone may therefore underestimate clinically relevant subsurface damage. These findings are hypothesis-generating and should not be considered definitive. Intraoral OCT may complement routine IOS by providing non-invasive, chairside information on subsurface damage in CAD/CAM composite restorations and may thereby support individualized recall scheduling and minimally invasive repair decisions. However, its added value for fatigue monitoring needs to be confirmed in larger in-vitro, clinical studies and across a broader range of restorative materials.

## Figures and Tables

**Table 1:** Material used in the study (information provided by manufacturer).

Code	Product Name	Manufacturer	Batch No.	Shade	Polishing System	Pre-Treatment	Bonding Agent	Luting System
BC	Brilliant Crios	Coltene	L67588	A2	DIATECH Finishing and Polishing Kit	Blasting with aluminum oxide powder (50 μm, 1.5 bar)	One Coat 7 Universal	DuoCem

**Table 2:** Descriptive statistics for intraoral scans (IOS) and optical coherence tomography (OCT) derived metrics at each timepoint, reported as mean ± standard deviation (SD) and median (interquartile range (IQR)).

	Timepoint	Vertical		Horizontal	
		Mean ± SD	Median (IQR)	Mean ± SD	Median (IQR)
IOS					
	T1	4.36 ± 0.91	4.13 (1.03)	13.71 ± 1.28	13.92 (1.78)
	T2	5.33 ± 1.47	5.33 (2.12)	17.68 ± 2.29	18.21 (1.44)
	T3	6.17 ± 1.36	6.07 (2.07)	19.63 ± 0.97	19.94 (0.82)
	T4	7.32 ± 1.22	7.50 (1.63)	20.71 ± 1.44	20.71 (1.35)
	T5	7.78 ± 3.19	8.43 (2.93)	21.06 ± 1.18	20.68 (1.36)
OCT					
	T1	25.47 ± 4.97	26.73 (5.88)	25.19 ± 3.99	25.30 (5.76)
	T2	37.15 ± 8.88	37.45 (11.54)	28.66 ± 2.07	29.54 (3.78)
	T3	44.67 ± 12.17	48.14 (17.41)	33.14 ± 3.24	33.12 (5.36)
	T4	59.44 ± 13.99	63.86 (14.54)	38.95 ± 4.99	39.16 (1.87)
	T5	66.79 ± 19.53	69.52 (15.47)	38.42 ± 3.67	37.70 (3.13)

Timepoints: After 250,000 (T1), 500,000 (T2), 750,000 (T3), 1,000,000 (T4), and 1,250,000 (T5) load cycles.

**Table 3:** Spearman’s correlation coefficient (ρ) and *p*-value between external surface wear and subsurface damage measurements.

	Timepoint	ρ	*p*-Value
vertical			
	T1	−0.024	0.955
	T2	0.419	0.301
	T3	0.563	0.146
	T4	0.381	0.352
	T5	0.434	0.283
horizontal			
	T1	0.071	0.867
	T2	−0.238	0.570
	T3	0.048	0.911
	T4	0.024	0.955
	T5	−0.429	0.289

Timepoints: After 250,000 (T1), 500,000 (T2), 750,000 (T3), 1,000,000 (T4), and 1,250,000 (T5) load cycles.

## Data Availability

The datasets in this article are available from the corresponding author upon reasonable request.

## Abbreviations

BC	Brilliant Crios
CAD/CAM	Computer-aided design/computer-aided manufacturing
CNC	Computer-numerical controlled
IOS	Intraoral scan
OCT	Optical coherence tomography
SS	Swept-source
